# Effect of four classes of antihypertensive drugs on cardiac repolarization heterogeneity: A double-blind rotational study

**DOI:** 10.1371/journal.pone.0230655

**Published:** 2020-03-24

**Authors:** Heini Sánez Tähtisalo, Timo P. Hiltunen, Tuomas Kenttä, Juhani Junttila, Lasse Oikarinen, Juha Virolainen, Kimmo K. Kontula, Kimmo Porthan

**Affiliations:** 1 Department of Medicine, University of Helsinki and Helsinki University Hospital, Helsinki, Finland; 2 Research Program for Clinical and Molecular Metabolism, Faculty of Medicine, University of Helsinki, Helsinki, Finland; 3 Research Unit of Internal Medicine, Medical Research Center Oulu, Oulu University Hospital and University of Oulu, Oulu, Finland; 4 Division of Cardiology, Heart and Lung Center, University of Helsinki and Helsinki University Central Hospital, Helsinki, Finland; 5 Department of Medicine, University of Helsinki and Minerva Foundation Institute for Medical Research, Helsinki, Finland; University of Messina, ITALY

## Abstract

**Background:**

T-wave area dispersion (TW-Ad) is a novel electrocardiographic (ECG) repolarization marker associated with sudden cardiac death. However, limited data is available on the clinical correlates of TW-Ad. In addition, there are no previous studies on cardiovascular drug effects on TW-Ad. In this study, we examined the relation between TW-Ad and left ventricular mass. We also studied the effects of four commonly used antihypertensive drugs on TW-Ad.

**Methods:**

A total of 242 moderately hypertensive males (age, 51±6 years; office systolic/diastolic blood pressure during placebo, 153±14/100±8 mmHg), participating in the GENRES study, were included. Left ventricular mass index was determined by transthoracic echocardiography. Antihypertensive four-week monotherapies (a diuretic, a beta-blocker, a calcium channel blocker, and an angiotensin receptor antagonist) were administered in a randomized rotational fashion. Four-week placebo periods preceded all monotherapies. The average value of measurements (over 1700 ECGs in total) from all available placebo periods served as a reference to which measurements during each drug period were compared.

**Results:**

Lower, i.e. risk-associated TW-Ad values correlated with a higher left ventricular mass index (*r* = −0.14, *p* = 0.03). Bisoprolol, a beta-blocker, elicited a positive change in TW-Ad (*p* = 1.9×10^−5^), but the three other drugs had no significant effect on TW-Ad.

**Conclusions:**

Our results show that TW-Ad is correlated with left ventricular mass and can be modified favorably by the use of bisoprolol, although demonstration of any effects on clinical endpoints requires long-term prospective studies. Altogether, our results suggest that TW-Ad is an ECG repolarization measure of left ventricular arrhythmogenic substrate.

## Introduction

Despite recent great progress in cardiovascular disease prevention, sudden cardiac death (SCD) remains a major contributor to mortality, accounting globally for 1.5 million deaths yearly [1]. In fact, up to 20% of all deaths have been estimated to occur unexpectedly, most likely due to ventricular fibrillation or asystole [2]. Due to the major impact of SCD at the population level, a number of predictive risk stratification techniques have been proposed, including analyses of autonomic nervous system function, cardiac imaging, molecular genetic assays as well as electrocardiographic (ECG) analyses [2–4] but none has proven to fully satisfy requirements for population screening.

Methods based on ECG analyses could provide a readily available means for SCD risk stratification. A large number of potential markers for electrical instability have been evaluated, including heart rate and its variability, QRS duration, QT interval, QT dispersion, early repolarization, J-wave heterogeneity, and T-wave abnormalities such as T-wave peak to T-wave end (TPE) interval, T-wave alternans, and T-wave heterogeneity [2,5]. A cumulative ECG risk score assessing ≥4 ECG abnormalities simultaneously was recently found to improve risk classification in a community-based study [6]. Very recently, a new marker for repolarization heterogeneity, T-wave area dispersion (TW-Ad), was proposed and shown to be a powerful and independent predictor of SCD in an adult general population sample [7].

A number of commonly used drugs, especially antimicrobial, antiarrhythmic, antipsychotic, and antidepressant drugs, have been found to interfere with cardiac repolarization mechanisms and thus provoke life-threatening arrhythmias [8]. In this respect, there should be particular interest in antihypertensive drugs since these are widely used in cardiac patients. Originally designed for pharmacogenomic studies, our GENRES study should offer an ideal opportunity for detailed studies on antihypertensive drug effects on cardiac electrical properties. In GENRES, over 200 hypertensive subjects were subjected to four different types of antihypertensive monotherapies (a diuretic, beta-blocker, calcium channel blocker, angiotensin receptor antagonist) in a double-blind and rotational fashion [9]. In the present study, we report the association between TW-Ad and echocardiographic left ventricular mass as well as the effects of the four antihypertensive drugs on this novel cardiac ECG repolarization parameter.

## Methods

The GENRES study was approved by the Ethics Committee of Helsinki University Central Hospital and the National Agency for Medicines of Finland. The clinical part of the study was carried out at the Helsinki University Central Hospital during 1999–2004 in accordance with the Declaration of Helsinki and Guidelines for Good Clinical Practice (1996). All subjects gave a signed informed consent prior to study activities. The study is registered at ClinicalTrials.gov (NCT03276598).

The GENRES study was a randomized, double-blind, cross-over and placebo-controlled study that has been described previously in detail [9]. Briefly, 313 Finnish males aged 35–60 years were initially screened. Subjects with hypertension, defined as use of antihypertensive medication or diastolic office blood pressure (BP) ≥95 mmHg on 3 separate measurements, were included in the study. Exclusion criteria included use of ≥3 antihypertensive drugs prior to the study, secondary hypertension, body mass index >32 kg/m^2^, drug-treated diabetes mellitus, coronary heart disease, congestive heart failure, serum creatinine >115 μmol/L, clinically significant liver disease, alcohol/drug abuse, cerebrovascular disease, obstructive pulmonary disease and a disease treated with corticosteroids. During the study, the subject was withdrawn if any of the following was observed: normotension, noncompliance, an exclusion criterion, BP ≥200/120 mmHg, clinically significant echocardiographic finding [significant left ventricular hypertrophy (LVH), aortic valve insufficiency, dilated aortic root, old infarction scar], or other clinically significant medical condition as judged by the study physician. Data collected before withdrawal were used in the analyses for those who were withdrawn during their ongoing study. Furthermore, the subject was excluded from this ECG study if he had a complete bundle branch block in ECG, or no placebo ECG. After exclusions, a total of 242 subjects were included in this ECG study.

The treatment phase of the study consisted of eight 4-week periods (placebo or active monotherapy drug) (S1 Fig). The first period was a 4-week placebo period. Thereafter, periods alternated between monotherapy and placebo, resulting in a total treatment phase of 8 × 4 = 32 weeks. Possible previous antihypertensive medication was discontinued before the beginning of the first placebo period. Tests taken at the end of the first placebo period were defined as baseline tests. BP, ECG and blood samples were taken at the end of each study period. During the active monotherapy drug periods, all subjects used in a randomized order an antihypertensive drug: amlodipine 5 mg, bisoprolol 5 mg, hydrochlorothiazide (HCTZ) 25 mg, or losartan 50 mg (daily oral doses for each).

### Electrocardiography

A digital standard resting 12-lead ECG was recorded at the end of all eight study periods with a Marquette MAC 5000 electrocardiograph (GE Marquette Medical Systems, Milwaukee, WI). QT Guard 1.3 software (GE Marquette Medical Systems) produced automatically digital median QRS-T complexes for all 12 leads. ECG LVH was considered to be present if (with 10mm/mV calibration) Sokolow-Lyon voltage, defined as S_V1_ + R_V5_ or R_V6_ (whichever was greater) >35 mm or Cornell product, defined as (S_V3_ + R_aVL_) × QRS duration >2440 mm×ms was present [10].

ECG repolarization parameters associated with SCD in previous studies were selected to compare their correlation with TW-Ad. The selected parameters were QT interval [11,12], TPE interval [13], T-wave morphology dispersion (TMD) [14], total cosine R-to-T (TCRT) [14], and T-wave residuum [14]. All ECG measurements were performed blinded to clinical data, and were reviewed onscreen by a researcher. TW-Ad, a measure of ventricular repolarization heterogeneity, was determined with custom-made software as described previously [7]. TW-Ad is an average of normalized areas from QRS end to T-wave end and varies between −1 and 1. Values closer to 1 indicate less variability while lower values indicate more variability between T-waves in different leads, as described in Fig 1 of [7]. Leads I, II, and V_4_-V_6_ were used for calculating TW-Ad, and no heart rate correction was used, as recommended by Kenttä et al. [7]. For calculation of TW-Ad, leads I, II, and V_4_-V_6_ are suitable because T waves in them are normally upright, and T-wave inversions in them also have prognostic value [7]. QT interval (maximum QT interval of 12 leads), i.e. the sum of duration of ventricular depolarization and repolarization, was measured from QRS onset to T-wave end with a custom-made software. The nomogram method was used for heart rate adjustment of QT intervals [15,16]. TPE interval was measured from T-wave peak to T-wave end. Longer TPE intervals represent increased dispersion of ventricular repolarization [13]. V_5_ was used for TPE interval analyses. If TPE was not measurable in V_5_, V_4_ and V_6_ were used (in that order) [13]. TMD and TCRT were calculated automatically using custom-made software with an eight-lead (I, II, V_1_-V_6_) singular value decomposition method as described previously [17]. TMD is a measure of spatial morphologic variation between T-waves of different leads. Dissimilar T-waves between different leads result in greater TMD values and represent higher degree of repolarization abnormality. TCRT is determined as the cosine of the angle between depolarization and repolarization vectors. High TCRT value refers to a small angle between R- and T-wave loop vectors, which is seen when depolarization and repolarization phases are normal [17]. TWR is a measure of regional repolarization heterogeneity, where higher TWR values are expected to be harmful compared to lower values [18].

**Fig 1:**
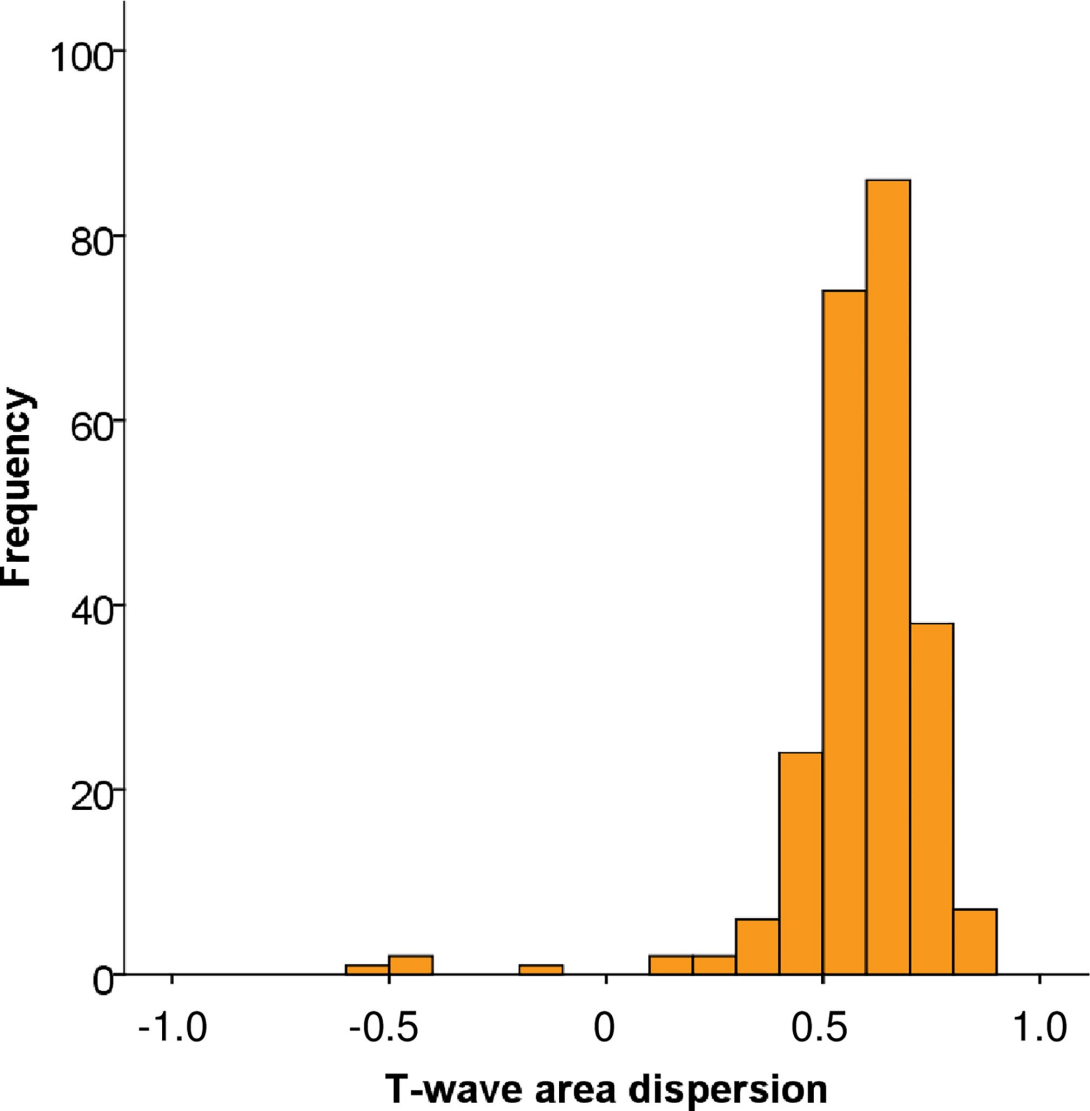
Distribution of mean T-wave area dispersion during placebo periods. For each subject, mean of up to four recordings was used.

### Echocardiography

Transthoracic echocardiography was performed at the end of the first placebo period by an experienced cardiologist. Echocardiographic information was available for 235 (97%) subjects. All echocardiographic measurements were averaged from five cardiac cycles. Left ventricular mass in grams was calculated from M-mode (n = 226) or 2-D (n = 9) recordings with an anatomically validated formula: 0.8 × [1.04 × ((interventricular septal thickness + left ventricular end-diastolic diameter + posterior wall thickness)^3^ –left ventricular end-diastolic diameter^3^)] + 0.6 [19]. Ejection fraction was calculated using the Teichholz method. Left ventricular mass index (LVMI) was indexed to body surface area, and 115 g/m^2^ was the cutoff for LVH [10].

### Statistics

SPSS version 22.0 (IBM SPSS Statistics, Armonk, NY) was used for the statistical analyses. Values are given as mean±SD, unless otherwise stated. The average value of measurements (BP and ECG data) from all available placebo periods served as a reference (later named as placebo) to which measurements during each drug period were compared. Differences between the effects of the four drugs on TW-Ad were assessed with the Friedman test. Individual drug effects on TW-Ad were tested with the Wilcoxon signed ranks test. Bivariate correlations between placebo TW-Ad (or drug-induced changes in TW-Ad) and selected variables were analyzed with Spearman’s test, since TW-Ad was nonnormally distributed, as judged by significantly negative skewness (test statistic -3.5 with a standard error of 0.16) and Kolmogorov-Smirnov test (*p* = 1.6×10^−15^). To identify independent determinants of placebo TW-Ad, we used multiple stepwise linear regression analysis with *p*<0.10 as an inclusion criterion (normalized TW-Ad values were used in this analysis). The tested covariates were LVMI, age, body mass index, serum creatinine, smoking, serum potassium, and 24-hour ambulatory and office systolic and diastolic BP levels. LVMI was divided in five categories of equal intervals, and the difference between LVMI categories was tested with the Jonckheere-Terpstra test. Two-tailed *p*<0.05 was considered significant in all analyses.

## Results

### Subject characteristics

Baseline characteristics of the subjects are presented in Table 1. Study sample comprised 242 moderately hypertensive middle-aged men. Of the analyzed potassium, calcium, creatinine, and glucose blood samples, 91% had results within laboratory reference range and there were no clinically significant abnormal values. No wall-motion abnormalities were seen with echocardiography. ECG LVH was present in 28 (12%) and echocardiographic LVH in 41 (17%) subjects.

**Table 1:** Characteristics of the study population.

Variables	All subjects (n = 242)
Clinical characteristics	
Age, years	50.5±6.4
Body mass index, kg/m^2^	26.7±2.8
Current smokers, n (%)	38 (16)
Office mean SBP during placebo periods, mmHg	153±14
Office mean DBP during placebo periods, mmHg	100±8
24-h mean SBP during placebo periods, mmHg	135±11
24-h mean DBP during placebo periods, mmHg	93±6
Laboratory tests ^a^	
Serum potassium, mmol/L	4.4±0.2
Serum calcium, mmol/L	2.38±0.08
Serum creatinine, μmol/L	87±13
Serum glucose, mmol/L	5.4±0.6
Total plasma cholesterol, mmol/L	5.6±0.9
Electrocardiographic parameters ^b^	
Heart rate, beats/min	62±8
QT interval, nomogram-corrected, ms	400±19
T-wave area dispersion, unitless	0.59±0.17
T-wave morphology dispersion, degrees	13.5±11
T-wave cosine R to T, unitless	0.41±0.45
T-wave residuum, technical units	15474±16278
Sokolow-Lyon voltage, mm ^c^	25±7
Cornell product, mm×ms ^c^	1438±531
Electrocardiographic left ventricular hypertrophy, n (%) ^d^	28 (12)
Echocardiographic parameters	
Ejection fraction, %	62±7
Left ventricular mass index, g/m^2^	98±19
Echocardiographic left ventricular hypertrophy, n (%) ^e^	41 (17)

Values are expressed as mean±SD, or numbers and percentages. DBP indicates diastolic blood pressure; SBP, systolic blood pressure.

^a^Fasting values taken after the first placebo period.

^b^Mean of all placebo ECGs.

^c^Calibration 10mm/mV.

^d^ECG left ventricular hypertrophy was considered to be present if increased Sokolow-Lyon voltage or Cornell product was present.

^e^Left ventricular mass index >115 g/m^2^.

### TW-Ad during placebo treatment

Placebo ECG data was derived from four recordings in 187 (77.3%) subjects, from three recordings in 23 (9.5%) subjects, and from one or two recordings in the remaining 32 (13.2%) subjects. Mean placebo TW-Ad was 0.59±0.17, and it was nonnormally distributed (Fig 1). Only 12 (5.0%) of the subjects had TW-Ad values at the level that has previously been associated with an elevated risk of SCD (TW-Ad ≤0.46) [7]. Repeatability of TW-Ad was good, based on graphical evaluation (S2 Fig) and on the concise median within-subject range (0.12) during the placebo measurements.

TW-Ad correlated significantly with QT interval (*r* = −0.24, *p* = 1.5×10^−4^), TMD (*r* = −0.48, *p* = 5.0×10^−15^), TCRT (*r* = 0.38, *p* = 1.1×10^−7^), and TWR (*r* = −0.19, *p* = 0.01), but not with TPE interval (*r* = −0.05, *p* = 0.51) (S1 Table). There was also a significant correlation between TW-Ad and body mass index (*r* = 0.18, *p* = 0.005), as well as between TW-Ad and creatinine clearance values (*r* = 0.21, *p* = 0.001). No correlation was observed between TW-Ad and office or ambulatory BP levels.

### A negative correlation between TW-Ad and measures of left ventricular mass

**Fig 2:**
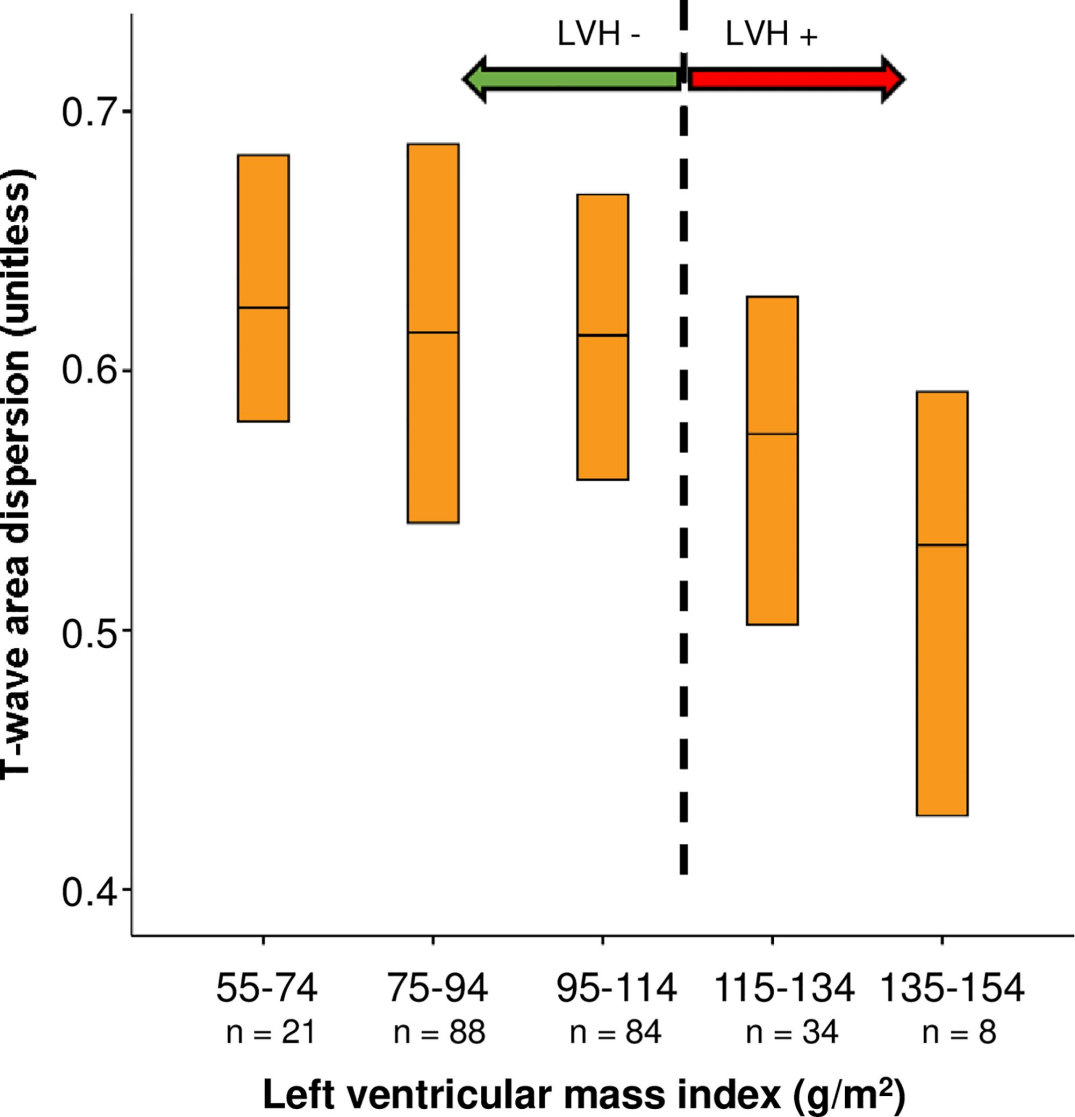
TW-Ad during placebo periods in five ordered left ventricular mass index categories. Medians and interquartile ranges are shown. *p* = 0.02 for trend (Jonckheere-Terpstra test). *p* = 0.01 for LVH- vs. LVH+ (Mann-Whitney U test). LVH indicates left ventricular hypertrophy.

Of the LVH-related ECG parameters, higher Sokolow-Lyon voltage was associated with lower TW-Ad values (*r* = −0.34, *p* = 7.3×10^−8^) but no association was observed between Cornell voltage duration product and TW-Ad (S1 Table). In addition, higher LVMI values correlated with lower TW-Ad values (*r* = −0.14, *p* = 0.03) (S1 Table). A significant linear trend (*p* = 0.02) for TW-Ad in five ordered LVMI categories was also observed (Fig 2). In multiple stepwise linear regression analysis, LVMI remained as a significant covariate (standardized β = -0.13, *p* = 0.03). Other significant covariates were body mass index (standardized β = 0.16, *p* = 0.006) and serum creatinine (standardized β = -0.14, *p* = 0.02) while age, serum potassium, smoking, and BP levels were non-significant.

### The effects of four antihypertensive drugs on TW-Ad

**Fig 3:**
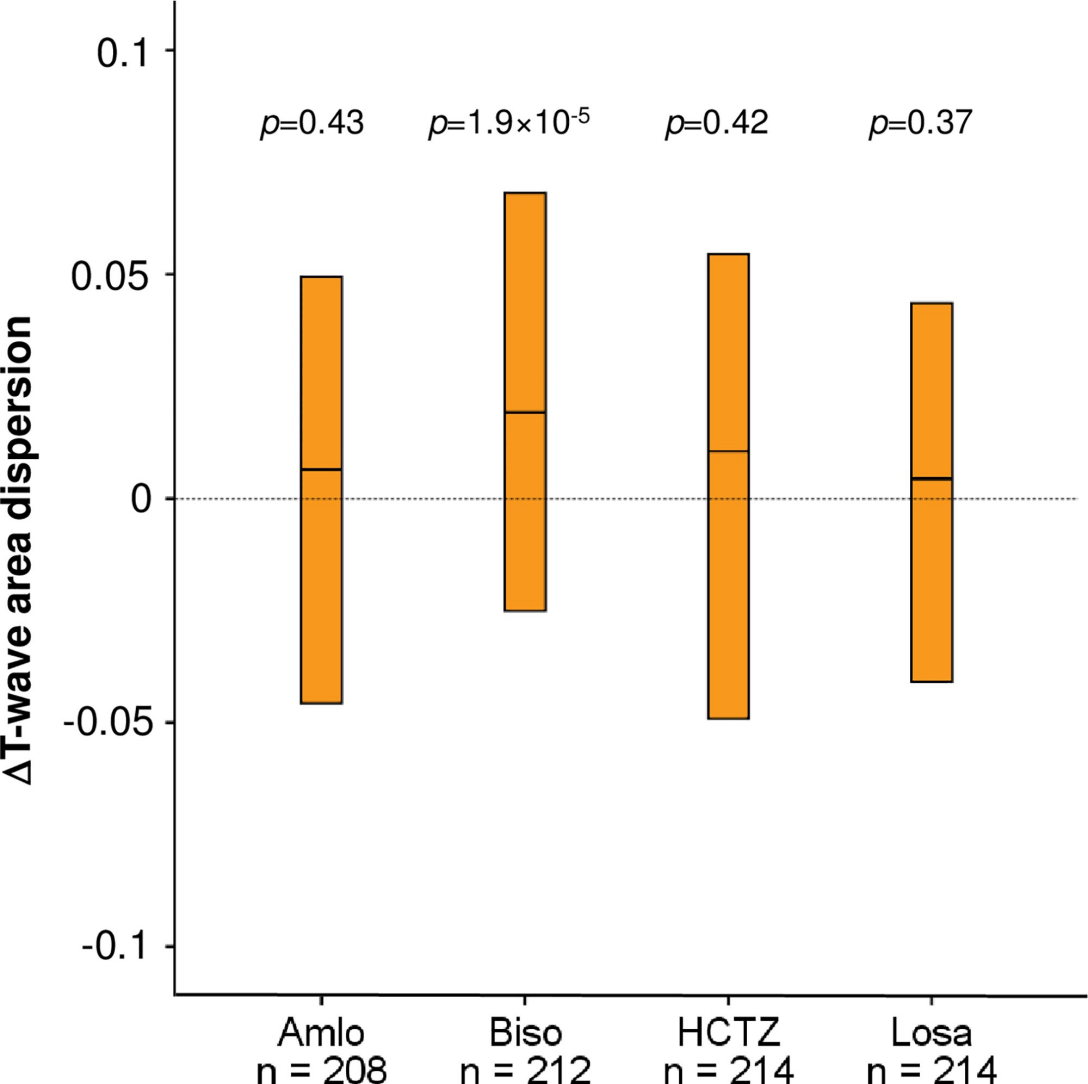
T-wave area dispersion changes after four different four-week antihypertensive monotherapies. Medians and interquartile ranges are shown. *p* values are for change from baseline values (Wilcoxon signed rank test). Amlo indicates amlodipine; Biso, bisoprolol; HCTZ, hydrochlorothiazide; Losa, losartan.

Four-week treatment with bisoprolol elicited a significant (*p* = 1.9×10^−5^) positive change in TW-Ad (Fig 3). A total of 127 (63%) of subjects had higher TW-Ad values on bisoprolol than on placebo, and the median change of TW-Ad was 0.02. In other words, the median TW-Ad on placebo, 0.61, increased during bisoprolol treatment to a median of 0.63. Amlodipine, HCTZ, or losartan did not cause significant changes in TW-Ad.

Change in TW-Ad during bisoprolol correlated with baseline age (*r* = −0.16, *p* = 0.02) and with baseline creatinine clearance (*r* = 0.15, *p* = 0.03). There was also a correlation between change in TW-Ad and change in Cornell product during bisoprolol (*r* = −0.22, *p* = 0.002) (S2 Table). However, change in TW-Ad during bisoprolol did not correlate with baseline LVMI or with BP change, heart rate change, QT-interval change, or with Sokolow-Lyon voltage change during bisoprolol (S2 Table).

## Discussion

In this study, we examined antihypertensive drug effects on TW-Ad, a novel repolarization marker, in moderately hypertensive middle-aged men. The most important findings were the relationship between higher left ventricular mass and unfavorable TW-Ad values, as well as the beneficial effect of bisoprolol treatment on TW-Ad.

Low TW-Ad values (≤0.46) were recently reported to predict SCD in two population-based cohorts [7]. In our GENRES cohort, where significant cardiac abnormalities and comorbidities were excluded, mean TW-Ad was 0.59, and only 8.7% of the subjects had TW-Ad values ≤0.46. However, even in this relatively healthy cohort, we observed an association between higher echocardiographic LVMI and lower, presumably more unfavorable TW-Ad values. Our results correspond well to our previous results, where associations between LVMI and other repolarization markers (QT interval, TPE, TMD, TCRT, TWR) were observed in the same GENRES cohort [16]. LVH is a known risk factor for ventricular tachyarrhythmias and SCD [20–22], presumably at least in part due to the associated repolarization abnormalities.

Baseline TW-Ad values during placebo treatment correlated with Sokolow-Lyon voltage but not with Cornell product in this study. The correlation between TW-Ad values and Sokolow-Lyon voltage estimates could result from their ability to monitor similar ECG changes, both taking V_5_ or V_6_ into account. In contrast, Cornell product and TW-Ad were not associated, possibly due to the different ECG leads used to calculate them. This may also be related to our study population consisting of only men because Cornell-based criteria produce markedly lower ECG LVH prevalence rates in men than in women [23]. In addition, some previous studies suggest that Sokolow-Lyon voltage and Cornell product evaluate different left ventricular qualities in echocardiography [24].

One of the principal findings of the present study was the presumably beneficial effect of bisoprolol on TW-Ad (Fig 3). Due to the relatively short treatment period, it is likely that drug effects on TW-Ad are mediated by direct pharmacological and as yet unknown mechanisms which may be other that those involving left ventricular mass reduction [25,26]. Beta-blockers have been previously demonstrated to significantly decrease the prevalence of ventricular arrhythmias after acute myocardial infarction [27]. Beta-adrenergic stimulation increases intracellular calcium ion concentration, which leads to delayed after depolarizations predisposing to arrhythmias. In addition, increased beta-adrenergic stimulation leads to increased potassium outward currents that shorten action potential duration, which also predisposes to arrhythmia [28,29]. Bisoprolol, as a beta-adrenergic antagonist, attenuates the initial steps in this chain of events thus lowering the possibility of tachyarrhythmia. The present data is in accordance with our earlier observations of the effect of bisoprolol on some other repolarization parameters, including QT interval, TPE interval, TMD, and TCRT [30]. These effects appear to be compatible with the antiarrhythmic effects of bisoprolol. Clinically, beta-blockers are known to reduce SCD events e.g. in long QT syndrome, catecholaminergic polymorphic ventricular tachycardia, heart failure, and myocardial infarction [31,32].

Other antihypertensive medications used in this study (amlodipine, HCTZ and losartan) did not induce significant changes in TW-Ad values during short-term treatment. In our previous study of drug effects on cardiac repolarization parameters, we found potentially favorable effects of losartan on QT interval, TPE and TMD, in contrast to HCTZ, which increased TPE [30].

The correlation between TW-Ad and other repolarization markers in the present study was somewhat variable. The strongest relationship was observed to TMD; both the TMD values during placebo treatment and the drug-induced changes in TMD were significantly correlated with the corresponding TW-Ad values. Correlations between TW-Ad and other parameters of T-wave morphology (TMD, TCRT, TWR) were also significant. Equal T-wave areas between leads result in a TW-Ad value close to 1. Even minor changes in T-waves between leads add diversity between T-wave areas resulting in smaller or even negative normalized areas, resulting in TW-Ad values closer to its most negative result, i.e. −1. Similarly, changes in T-waves between leads are a sign of repolarization heterogeneity, manifesting in elevated TMD and TWR values, and reduced TCRT values.

No correlation was detected between TW-Ad and QT interval values in the study introducing TW-Ad [7], whereas the present data indicated a significant inverse correlation between QT interval and TW-Ad values. In principle, this type of correlation is not unexpected, as T-wave constitutes a part of the QT interval. Bazett correction for the QT interval was used in the study by Kenttä et al. [7], while in the present study a nomogram correction derived from another all-male Finnish cohort [15] was employed. Accordingly, the differences in the observed TW-Ad vs. QT correlation in these two studies could be partly explained by the use of different QT correction formulas.

QT interval has been proposed as a risk stratifier for SCD [2], implying that a longer QT interval is associated with increased cardiac mortality. However, despite extensive studies the association between QT interval and SCD has remained somewhat controversial [3,33–35]. Further long-term mortality studies may be needed in which measurements of both QT interval and TW-Ad values are prospectively monitored.

On one hand, the results of the present study are to be interpreted in the light of certain limitations. The GENRES study subjects were moderately hypertensive Finnish males with no significant comorbidities, and the results may not be generalizable to women or subjects with other ethnicities or with significant comorbidities. Due to the complex design of the GENRES study, the size of the study cohort is unavoidably relatively small. Therefore, the power of the study may be too low for detection of small associations or effects, and some of the observed findings may have occurred by chance. Furthermore, the target parameter of this study, TW-Ad, is a surrogate and not a true clinical endpoint.

On the other hand, it should be emphasized that the GENRES study cohort has been meticulously phenotyped and offers major advantages for ECG research. Altogether, over 1700 ECGs were reviewed in a blinded manner for this study. In addition, the accuracy of the estimates of the baseline TW-Ad values is highlighted by the fact that they were mostly derived from four separate recordings during placebo treatment. Finally, it is also of note that the GENRES platform provides an excellent tool to investigate drug class specificity of any positive effects on repolarization parameters.

## Conclusions

Our results provide additional support for the use of TW-Ad as a repolarization parameter that can be reliably estimated in repeated occasions and is associated with indices of left ventricular mass. Beta-blocker bisoprolol exerts apparently favorable effects on TW-Ad values. These results suggest that TW-Ad is a measure of left ventricular arrhythmogenic substrate. However, further prospective and long-term studies are needed to clarify the exact role of TW-Ad measurements in risk assessment and individualized drug therapy in cardiovascular disease.

## Supporting information

S1 Table Correlations between T-wave area dispersion during placebo periods and selected baseline variables (significant *p* values are highlighted). (PDF)

S2 Table Correlations between changes in T-wave area dispersion and changes in selected variables during bisoprolol (significant *p* values are highlighted). (PDF)

S1 Fig GENRES study protocol. (PDF)

S2 Fig Repeatability of T-wave area dispersion. (PDF)
