# Supplementary material for: Effect of four classes of antihypertensive drugs on cardiac repolarization heterogeneity: A double-blind rotational study
Source: PLoS One. 2020 Mar 24;15(3):e0230655. doi: 10.1371/journal.pone.0230655 (PMC7092984; doi:10.1371/journal.pone.0230655)
Supplement: S1 Table — (PDF) [file pone.0230655.s001.pdf]

S1 Table. Correlations between T-wave area dispersion during placebo periods and selected baseline variables (significant *p* values are highlighted).

	Correlation with T-wave area dispersion	
	r	*p* value
**Clinical characteristics**		
Age	−0.12	0.07
Body mass index	0.18	0.01
Current smoking	−0.01	0.89
Office mean SBP during placebo periods	−0.01	0.87
Office mean DBP during placebo periods	0.01	0.89
24-h mean SBP during placebo periods	0.01	0.91
24-h mean DBP during placebo periods	0.03	0.69
**Laboratory tests**		
Serum calcium	0.12	0.06
Serum creatinine	−0.11	0.09
Creatinine clearance (Cockcroft-Gault)	0.21	0.001
Diurnal urinary albumin excretion	−0.14	0.03
Serum potassium	0.03	0.62
Fasting serum glucose	0.03	0.63
Total plasma cholesterol	0.04	0.59
**Electrocardiographic parameters**		
Heart rate	0.10	0.14
QT-interval, nomogram-corrected	−0.24	1.5×10^{-4}
T-wave morphology dispersion	−0.48	5.0×10^{-15}
T-wave peak to T-wave end interval	−0.05	0.51
Total cosine R-to-T	0.38	1.1×10^{-7}
T-wave residuum	−0.19	0.01
Sokolow-Lyon voltage	−0.34	7.3×10^{-8}
Cornell product	0.01	0.88
**Echocardiographic parameters**		
Ejection fraction	0.04	0.56
Left ventricular mass index	−0.14	0.03

DBP indicates diastolic blood pressure; SBP, systolic blood pressure.
