# Supplementary material for: Effect of four classes of antihypertensive drugs on cardiac repolarization heterogeneity: A double-blind rotational study
Source: PLoS One. 2020 Mar 24;15(3):e0230655. doi: 10.1371/journal.pone.0230655 (PMC7092984; doi:10.1371/journal.pone.0230655)
Supplement: S2 Table — (PDF) [file pone.0230655.s002.pdf]

S2 Table. Correlations between changes in T-wave area dispersion and changes in selected variables during bisoprolol (significant *p* values are highlighted).

	Correlation with Δ T-wave area dispersion	
	r	***p* value**
Δ Office mean SBP	−0.07	0.34
Δ Office mean DBP	−0.03	0.65
Δ 24-hour SBP	−0.08	0.28
Δ 24-hour DBP	−0.04	0.58
Δ Heart rate	−0.06	0.43
Δ QT interval	−0.08	0.27
Δ T-wave morphology dispersion	−0.45	1.4×10^{-10}
Δ Sokolow-Lyon voltage	−0.01	0.89
Δ Cornell product	−0.22	0.002

Δ indicates change; DBP, diastolic blood pressure; SBP, systolic blood pressure.
