# Supplementary material for: Effect of four classes of antihypertensive drugs on cardiac repolarization heterogeneity: A double-blind rotational study
Source: PLoS One. 2020 Mar 24;15(3):e0230655. doi: 10.1371/journal.pone.0230655 (PMC7092984; doi:10.1371/journal.pone.0230655)

S1 Fig. GENRES Study protocol.

All subjects used in a randomized order each of the four antihypertensive drug (red bars): amlodipine 5 mg, bisoprolol 5 mg, hydrochlorothiazide (HCTZ) 25 mg, or losartan 50 mg (daily oral doses for each). Four-week placebo periods (yellow bars) preceded all monotherapies.

24h ABP represents 24-hour ambulatory blood pressure; Drug 1, Drug 2, Drug 3, Drug 4, antihypertensive periods; ECG, electrocardiography; Echo, echocardiography; OBP, office blood pressure; P1, P2, P3, P4, placebo periods.

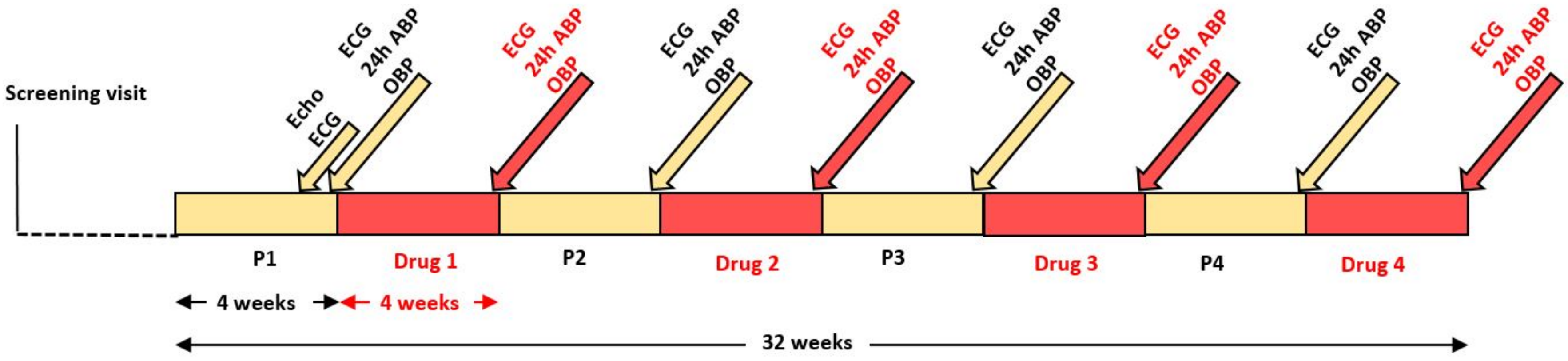

Supplement: S1 Fig — (PDF) [file pone.0230655.s003.pdf]
