# Supplementary material for: Effect of four classes of antihypertensive drugs on cardiac repolarization heterogeneity: A double-blind rotational study
Source: PLoS One. 2020 Mar 24;15(3):e0230655. doi: 10.1371/journal.pone.0230655 (PMC7092984; doi:10.1371/journal.pone.0230655)

S2 Fig. Repeatability of T-wave area dispersion.

Each dot represents a T-wave area dispersion (TW-Ad) value from one single placebo ECG (1−4 placebo ECGs per subject). Within-subject values are connected with a vertical line. Subjects are sorted on the horizontal axis according to their mean TW-Ad values. Median within-subject TW-Ad range was 0.12.

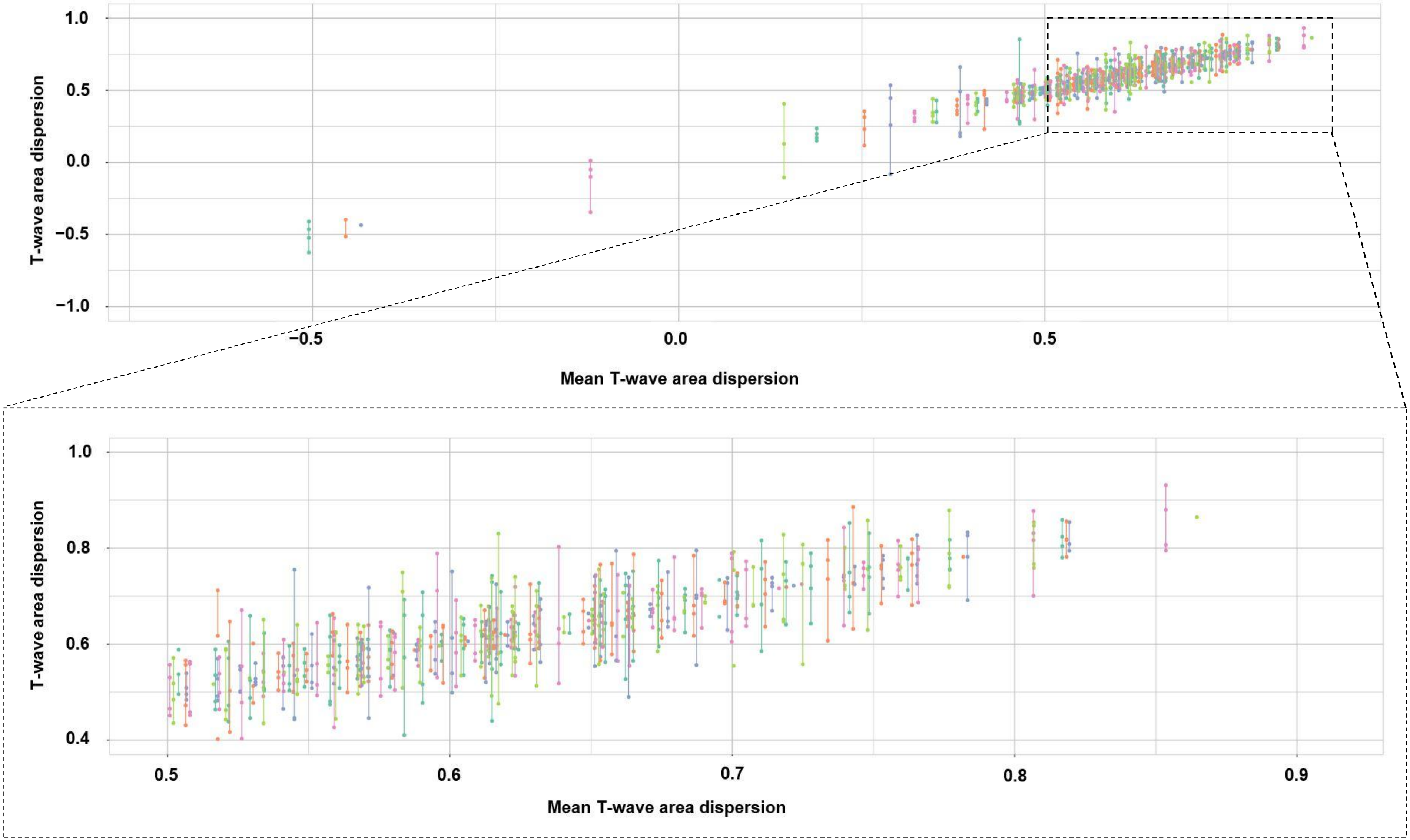

Supplement: S2 Fig — (PDF) [file pone.0230655.s004.pdf]
